# Seroepidemiology of chronic hepatitis B and C in the French Caribbean Island of Guadeloupe

**DOI:** 10.1186/1756-0500-7-55

**Published:** 2014-01-21

**Authors:** Moana Gelu-Simeon, Valérie Pillas, Jacqueline Deloumeaux, Hélène Delacroix-Maillard, Georgette Saint-Georges, Léonardo Do Amaral, Marius Borel, Marc Laurent, Emmanuel Gordien, Eric Saillard

**Affiliations:** 1 Department of Hepatology and Gastroenterology, University Hospital of Pointe-à-Pitre, Pointe-à-Pitre, Guadeloupe; 2 Research Group ECM, University Hospital of Pointe-à -Pitre, Pointe-à -Pitre, Guadeloupe; 3 Sainte Genevieve Health and Prevention Center, Pointe-à-Pitre, Guadeloupe; 4 Department of Virology, Avicenne Hospital, Bobigny, France

**Keywords:** Chronic hepatitis B, Chronic hepatitis C, Risk factors, French overseas territories, Caribbean island

## Abstract

**Background:**

The prevalence of chronic hepatitis B and C was evaluated some twenty years ago among specific populations in Guadeloupe. The present study was designed to update these data and determine epidemiological features of chronic hepatitis B and C infections in the French Caribbean island of Guadeloupe.

**Findings:**

The present study was carried out at the Sainte Genevieve Health and Prevention Center (Guadeloupe), between May 2006 and July 2007. This is a medical center where patients can attend a free medical check-up paid for by the Social Security system. Data on hepatitis B (HBV) and C (HCV) status and epidemiological factors were collected for this study.

A total of 2,200 patients were included in the study. The prevalence of HBV surface antigen was 1.41% (95% CI: 1.0-2.0), and 0.55% (95% CI: 0.28-0.96) for HCV. The vaccination rate against HBV was 42.0%. HBV transmission was associated with piercing (12.9%, p = 0.014) and familial exposure (6.4%, p < 0.001) and HCV transmission with gynecological surgery (50.0%, p = 0.01). The HBV profile was generally hepatitis B e antigen-negative (94.5%). No hepatitis delta was found. For HCV, genotype 1 was predominant (80%).

**Conclusions:**

This is the first study on the prevalence of HBV and HCV among a general clinic based population in Guadeloupe and the Caribbean islands. This study reveals that Guadeloupe is an area of low endemicity for HBV and low HCV prevalence. The reasons for these low prevalence rates are mainly related to the vaccination campaigns carried out during the past twenty years for HBV and the decrease of nosocomial transmission for HCV.

## Background

Guadeloupe, a French Caribbean island, has geographical and ethnic specificities, the majority population being Afro-Caribbean. The most recent scientific data on the prevalence of viral hepatitis infections in Guadeloupe were reported by Fest et al. [1,2] in 1993 among blood donors. They showed that hepatitis B surface antigen (HBsAg) was present in 3.1%, hepatitis B core antibodies (anti-HBc) in 22.1% and anti-hepatitis C virus antibodies (anti-HCV) in 0.8% of the population. Except for Puerto-Rico [3] and Haiti [4], which differ demographically and socioeconomically from the French Caribbean islands, seroprevalence data in the Caribbean region are scarce. Some studies have reported HCV genotype distribution in the Dominican Republic [5] and Puerto-Rico [6], but no information is available on the seroprevalence of hepatitis B and C in the Lesser Antilles archipelago.

## Methods

### Study population

The present study was carried out in Guadeloupe, a French department in the Caribbean, which counts 450,000 inhabitants. The Sainte-Genevieve Health and Prevention clinic is a social security medical center where beneficiaries of the health insurance system can attend for a free medical check-up. The Sainte-Genevieve Center is the only one Social Security-funded medical center in Guadeloupe, which is situated in Les Abymes, the principal urban community (home to a third of the Guadeloupean population). Between May and August 2006 and January and July 2007, beneficiaries of the French national health insurance system, aged over 18 years, were invited to undergo a free health check-up at the Sainte Genevieve Health and Prevention Center. They were informed of our study and invited to take part. This check-up included laboratory analyses and medical tests (vision, hearing, breathing capacity), supplemented by a physical examination by a physician. The investigations could be modulated according to items listed on the questionnaire (independent of our study), which was sent to these insured individuals when they made their appointments; these changes depended on the risk factors specific to their situation. We added serological screening for viral hepatitis and a questionnaire on the risk factors for hepatitis.

The initial questionnaire containing predetermined questions on medical history and sociodemographic data was completed by the patient him/herself. A second predetermined medical questionnaire focused on possible risk factors for HBV and/or HCV infections was then completed by the medical investigator during a face-to-face interview.

Our study was performed in accordance with the Declaration of Helsinki and was approved by a French Ethics Committee (*Comité Consultatif de Protection des Personnes dans la Recherche Biomédicale)* on May 20, 2003, n°03-251. All patients gave their written informed consent prior to inclusion in the study.

### Laboratory analyses

Serological tests for HBV and HCV were performed on all samples using AxSYM HBsAG (V2) for HBsAg, AxSYM CORE for anti-HBc, AxSYM AUSAB for anti-HBs antibodies and AxSYM HCV version 3.0 for anti-HCV antibodies (Abbott Laboratories, Chicago, IL, USA). The Monolisa anti-HCV plus (V2) from Biorad Company was used in the event of any doubtful results. Delta serology was assessed using the ETI 2AB deltaK2 kit obtained from Sorin. The tests were performed in strict accordance with the manufacturer’s instructions.

HBV DNA testing was performed using b-DNA HBV DNA version 3.0 (limit of detection: 2,000 copies/ml) and the HCV RNA technique b-DNA HCV RNA version 3.0 (limit of detection: 3,200 copies/ml) (Bayer Laboratories), according to the manufacturer’s recommendations (Bayer Diagnostics, Inc.).

HCV genotyping was performed by sequencing the NS5B region, followed by the phylogenetic analyses as described in reference 16. Biochemical tests on liver function (ALT, γGT), blood counts (hemoglobin, leukocytes), total cholesterol, triglycerides, serum creatinine and blood sugar were also performed.

### Statistical analysis

Statistical data were collected using Epi DATA and analyzed with STATA and SPSS 11.01. Dichotomous data were compared using the χ2 test α = 5%, or Fisher’s exact test when appropriate. Quantitative data were compared using Student’s t test. Exact Poisson confidence intervals were calculated.

## Findings

### Baseline characteristics of the study population

In 2006, 8,584 patients were seen at the health center, compared to 7,156 in 2007. As the investigating physician was present at the health center every two weeks in 2007 (versus weekly in 2006), 1,896 patients were eligible in 2006 and 1,576 in 2007. Out of the 3,472 patients who met the inclusion criteria, informed consent and complete data were obtained for 2,200 (63.3%) (Table 1). A significantly higher number of women participated (59.7% *vs.* 40.3%, p = 0.001) and the median age was 43 years [18–87]. The majority of the study population were of low socioeconomic status, the unemployment rate among them being 56.7%. A history of surgery (28.1%) or endoscopy (27.5%) was more frequently reported as a potential source of infection from HBV and/or HCV. The results of laboratory tests were all available and were within normal ranges.

**Table 1:** Baseline characteristics of the study population

***Characteristic***	***N = 2200 (%)***
***Age (median) years***	43 [18–87]
***Sexe***	
***Male***	886 (40.3)
***Female***	1314 (59.7)
***Occupation***	
***Unemployed***	1247 (56.7)
***Working class***	508 (23.1)
***Farmers***	103 (4.7)
***Middle/senior management***	125 (5.7)
***Retired***	217 (9.9)
***Risk factors***	
***Past surgery***	619 (28.1)
***Endoscopy***	584 (27.5)
***Using drugs***	13 (0.6)
***Intravenously***	1 (0.0)
***Nasal way***	12 (0.6)
***Tatoo***	216 (10.0)
***Piercing***	95 (4.4)
***Shaving hair or beard***	352 (16.3)
***Unknown***	321 (14.6)

### Chronic hepatitis B

A total of 31 patients (1.41%) (95% CI: 1.0%-2.0%) were positive for HBsAg and 594 (26.5%) (95% CI: 24.7%-28.4%) were positive for anti-HBc. 12 patients (38.7%) had PCR-detectable DNA (median value: 4.43 log copies/ml [3.32-8.00]).

Only 25% of patients had been aware of their serological status before testing. Most patients (94.5%) were HBeAg-negative. All subjects who were positive for HBs antigen were tested for hepatitis delta virus, and 100% of them were delta negative. The presence of HBsAg was three to four times more frequent in men than in women in the 30–39 and 60–69 years age groups. However, considering the overall HBsAg positive population, no significant difference between men and women (2.0% vs. 1.1%, NS) was found. HBV transmission was associated with one, two or three risk factors in 11, 7 and 4 cases respectively, and none in the remaining 9 patients (Table 2). Among these, piercing (12.9%, p = 0.014), familial exposure (6.4%, p < 0.001), and a history of surgery or endoscopy were more frequently reported.

**Table 2:** Risk factors of chronic hepatitis B transmission for positive HBsAg patients

***Risk factor***	***Positive HBs Ag patients***	***Negative HBsAg patients***	***P value***
	***N = 31 (%)***	***N = 2169 (%)***	
***Past surgery***	12 (38.7)	607 (28.0)	*NS*
***Endoscopy***	12 (38.7)	572 (26.4)	*NS*
***Piercing***	4 (12.9)	91 (4.2)	*p = 0.014*
***Shaving***	4 (12.9)	348 (16.0)	*NS*
***Tatoo***	3 (9.7)	213 (9.8)	*NS*
***Familial exposure***	2 (6.4)	33 (1.5)	*p < 0.001*
***Using drugs intraveinously***	0 (0.0)	1(0.0)	*NS*

*NS*, Not significant.

On the other hand, in the population study, the rate of vaccination against HBV (at least one injection) was 42.7% (95% CI: 40.6%-44.9%). Among them, 56.2% (95% CI: 54.1%-58.3%) had anti-HBs antibody titers higher than 20 IU/L.

### Chronic hepatitis C

Anti-HCV was detected in 14 out of 2,200 patients (0.55%; 95% CI: 0.28%-0.96%). The risk factors associated with HCV transmission are presented in Table 3. Three out of 14 patients reported no risk factor, while one or two risk factors were recorded for five and six patients, respectively (mainly a history of surgery or endoscopy). HCV-positive serological findings were significantly associated with previous surgery (57.1%, p = 0.02). It is noteworthy that two cases of transfusion were reported.

**Table 3:** Risk factors of HCV transmission

	***Positive HCV serology***	***Negative HCV serology***	***P value***
	***N = 14 (%)***	***N = 2186 (%)***	
***Past surgery including***	8 (57.1)	611 (27.9)	*p = 0.02*
***Gynaecological***	7 (50.0)	343 (15.7)	*p = 0.01*
***Endoscopy***	4 (28.5)	580 (26.5)	*NS*
***Tatoo***	2 (14.3)	214 (9.8)	*NS*
***Shaving***	1 (7.1)	351 (16.1)	*NS*
***Familial exposure***	0 (0.0)	17 (0.8)	*NS*
***Using drugs intraveinously***	0 (0.0)	1 (0.0)	*NS*
***Piercing***	0 (0.0)	95 (4.3)	*NS*
***Unknown***	3 (21.4)	318 (14.5)	*NS*

*NS*, Not significant.

Only one patient (7.1%) was aware of his positive serological status prior to the study. Ten patients (71.4%) had PCR-detectable RNA (median value: 6.36 copies/ml [5.91-6.86]) with 8/10 (80%) genotype 1 and 2/10 (20%) genotype 3.

## Discussion

This seroprevalence study of HBV and HCV was performed at the Sainte Genevieve Health and Prevention Center in order to update the data on chronic hepatitis in the general population of Guadeloupe. Seroprevalence was estimated at 1.41% for chronic HBV infection and 0.55% for HCV. The main risk factors were piercing, familial exposure for HBV and nosocomial exposure for HCV.

The Sainte Genevieve clinic is located in the main suburb of Guadeloupe, home to one-third of the island’s entire population. However, the estimated prevalence rates may not accurately reflect the overall prevalence of these infections on the island because the patient population is not demographically representative. Perhaps that periodically monitoring the prevalence of these infections in this clinic population could be a simple and effective approach for tracking HBV and HCV prevalence over time.

The only previous epidemiological study of HBV infection in Guadeloupe, reported by Fest et al. [2] in blood donors during the period 1992–1993, found a prevalence of more than 3.1%. The HBV prevalence of 1.4% seen in the present study among the clinic patients from 2006 suggests that the frequency of HBV infection in Guadeloupe may have decreased over time. This change might reflect vaccination campaigns that were carried out during this period. In a Guadeloupean study of a teenage population, it was found that 76.2% were vaccinated against HBV [7]. The percentage of patients consulting the center who were of low socioeconomic status was estimated at 49% in 2006 and 45% in 2007, and included the unemployed and those with minimal resources. Although unemployment affected 27% of the Guadeloupean population during the same period [8]. However, most patients residing in Guadeloupe are covered by health insurance that enables them to consult physicians in a hospital or private medical center rather than in a center run by the social security system. The sexual transmission of HBV was not studied here but it has been shown that in countries with a moderate prevalence of chronic HBV (1-5%), this rate is correlated to that of sexually transmitted diseases [9]. Shaving by a barber was described as the mode of HBV transmission in four HBsAg positive patients. This transmission route was also mentioned in an Italian study performed between 1997 and 2002 and which focused on beauty treatments and the routes of HBV transmission [10]. It revealed a statistically significant relationship between barber shaving and HBV transmission. It is possible that this factor lacked significance in our study because of the small numbers involved, but it would be interesting to include this factor in a larger population.

In summary, this study provides the first data concerning HBV prevalence in the general population of Guadeloupe, and reached a level of nearly 1.5%.

Concerning chronic hepatitis C, we found an overall prevalence of 0.55%. This low rate may be related to the modes of transmission. Indeed, the main mode of transmission was nosocomial in Guadeloupe (77.4%) and no intravenous drug injection transmission was determined.

Most of the subjects were infected with HCV genotype 1, probably related to the surgical transmission route, because the link between genotype 1b and hospital-based transmission is generally acknowledged [11]. Martial et al. [12] also reported genotype 1 as being predominant in Martinique HCV patients, essentially due to transmission via blood transfusions. Such a pattern of HCV transmission is unique. Indeed, in the French study reported by Meffre C et al., nearly 50% of HCV transmission was found in intravenous drug users and only 15% was transmitted nosocomially [13].

## Conclusions

This is the first study of the prevalence of chronic hepatitis B and C to have been performed in the general population of the French Caribbean island of Guadeloupe, without any distinctions being made with respect to age, gender or blood donor status. In addition to the high prevalence of hepatitis B infection, some of the epidemiological features of chronic hepatitis B and C, as well as their modes of transmission, were relevant. It would be useful to study these characteristics in a large Caribbean cohort from the population affected by chronic hepatitis. Concerted, island-wide efforts are necessary to reinforce prevention and control strategies to target HBV and improve surveillance. In this way, it will be possible to monitor hepatitis B infection and ensure the appropriate testing and medical management of infected individuals. Such measures would enable improvements to health practices and prevention in this part of the world.

## Abbreviations

HBV: Hepatitis B virus; HCV: Hepatitis C virus; HBsAg: Hepatitis B surface antigen; anti-HBc: Hepatitis B core antibodies; anti-HCV: Hepatitis C virus antibodies; anti-HBs: Hepatitis B surface antibodies; HBeAg: Hepatitis B e antigen; ALT: Alanine aminotransferase; γGT: Gamma-glutamyl transferase; HIV: Human immunodeficiency virus.

## Competing interests

The authors declare that they have no competing interest.

## Author’ contributions

MGS and EG performed the statistical analysis and drafted the manuscript. VP and ES performed the physical examinations and participated in the design and analysis of the study. JD performed the statistical analysis. HDM assisted with patient recruitment, and participated in analyzing the data. GSG, LA, MB and ML all participated in analyzing the data. All the authors read and approved the final manuscript.
